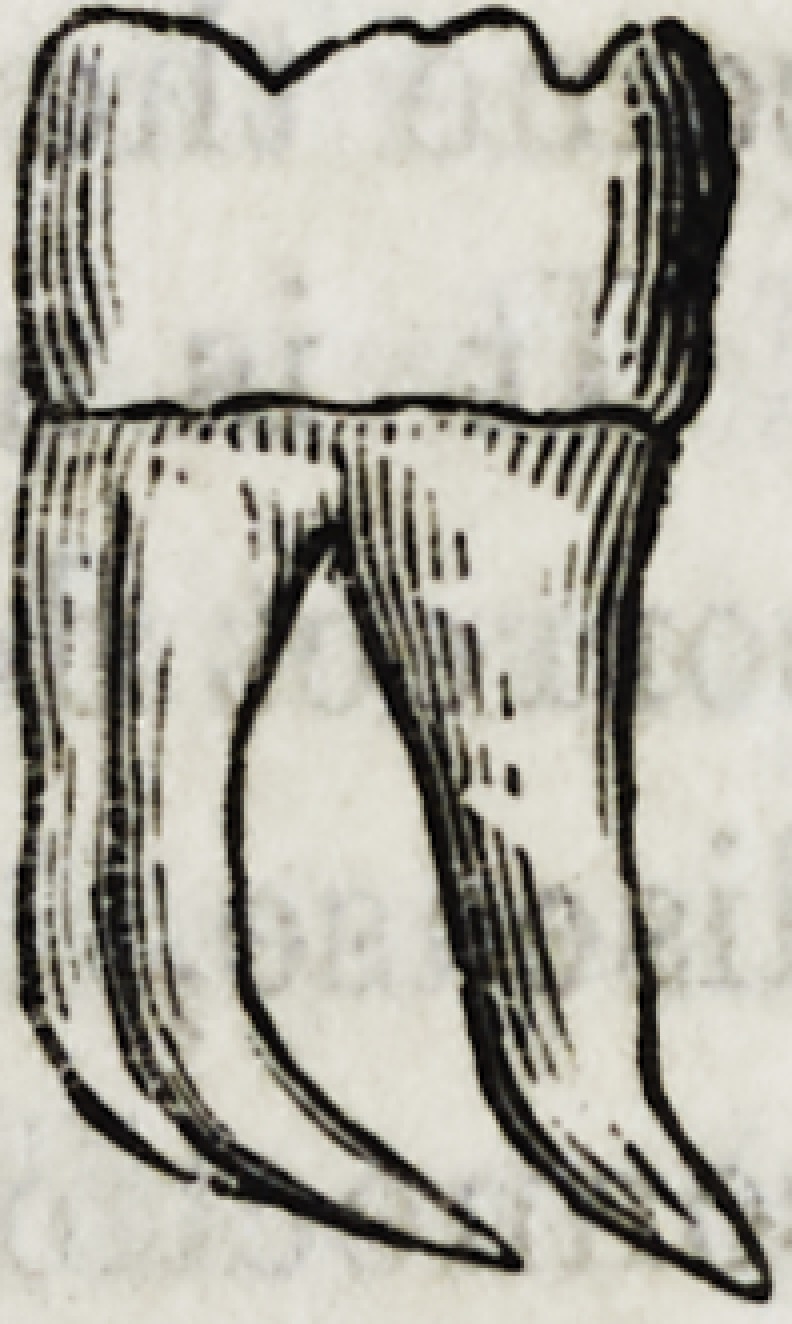# Tumor of Two Years Standing Cured by Extraction of Wisdom Tooth

**Published:** 1853-10

**Authors:** F. C. Scott

**Affiliations:** Dentist, Swansea, England.


### ARTICLE VII.

*Tumor of two years standing Cured by Extraction of Wisdom Tooth.* By F. C. Scott, Dentist, Swansea, England.

John Griffiths, aged 20, two years and a half since, whilst living about nine miles from Swansea, suffered from a slow fever, which greatly debilitated him. During his illness the dentes sapientiæ of the left side made their appearance, prior to which and afterwards he had severe pain on that side of the head, or to use his own words, "a violent face-ache." The pain was more acute in the inferior jaw, and the gum from the first molar to the wisdom tooth was swollen and greatly inflamed. On being lanced, it emitted a considerable quantity of pus, and the pain lessened but did not entirely subside. A few months after, the whole side of his face swelled to a considerable extent, and so continued, notwithstanding the use of a lotion, which his medical attendant prescribed for him. About this time, (December, 1851,) he obtained a situation in a shop in this town, and the swelling having assumed a hard appearance, he applied to me. A surgeon here, who gave him an ointment, (what I could not discover from his description,) to rub on the side of the face, telling him at the same time that it was "a growth of the bone," and that in his opinion the swelling would never entirely disappear. After using the ointment for about six months, without any success, he determined to seek further advice, for, besides the annoyance, being naturally good looking, the idea of one side of his face being nearly twice the size of the other was the reverse of pleasing, so he consulted Dr. ——, a physician of this town, who immediately lanced it. A slight quantity of pus escaped, the swelling diminished, but matter was discharged continually, the incision being kept open for nearly three months. At this period the pain (from which he had never been entirely free) began to increase, especially in the superior jaw, and after a week of great suffering he called on me for the first time to ask my advice.

On examination of the mouth I found that he had not the slightest symptom of caries in any of his teeth, nor had he ever had one extracted, both jaws presenting the full complement; on closer inspection, however, I discovered that the wisdom tooth of the superior jaw was much misshapen, the crown presenting a triangular appearance instead of a square. I was fully convinced the pain arose from undue pressure, and consequently advised the extraction of both the wisdom teeth on that side, to which, however, he would not consent; but the next day the pain being worse, he came and asked me to remove the upper dens sapientiæ, which I did. Annexed is a sketch of the tooth, the fangs being much curved and excessively sharp. Almost immediately after the removal of the tooth the pain ceased, but I could not then persuade him to let me extract the inferior wisdom tooth, which I strongly advised, for having been under the care of three medical men for nearly two years, with scarcely any relief, his face presenting an unsightly appearance, with a continual discharge, and as the removal of the tooth from the superior jaw had expelled the pain from that region, why might not the lower tooth have something to do with the tumor? However, another month passed by, and there being no sign of his face assuming a healthier aspect, Dr. —— recommended him to take my advice, and on January 4th, 1853, I extracted it. A slight quantity of pus escaped into the mouth on the removal of the tooth, which did not present any peculiar appearance besides the fangs being crooked posteriorly, as in sketch.

It is now nearly seven months since I extracted the tooth, the side of the face is restored to its original contour, and with the exception of the slight scar from lancing, nothing is discernible of the swelling that continued for upwards of two years, nor has he had the slightest symptom of pain since.

Velpeau relates a case of twenty months standing, where the patient could not open his mouth, and the tumefaction extended over the whole side of the face and neck. After forcing the mouth open, and keeping it so with cork and wood, the dens sapientiæ was extracted, and in six days after a piece of bone was discharged, the second molar was also extracted and a piece of bone again was removed, which had prevented the wisdom tooth from occupying its normal position. In less than three weeks he was quite restored, and his face had resumed its proper appearance. Mr. Tomes also relates many very instructing cases in his lectures on dental physiology and surgery.

## Figures and Tables

**Figure f1:**
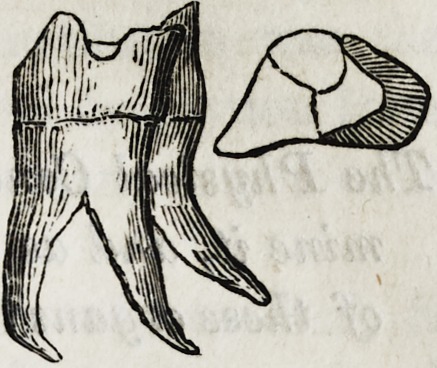


**Figure f2:**